# Climate change will influence disease resistance breeding in wheat in Northwestern Europe

**DOI:** 10.1007/s00122-021-03807-0

**Published:** 2021-03-13

**Authors:** Thomas Miedaner, Peter Juroszek

**Affiliations:** 1 State Plant Breeding Institute, University of Hohenheim, 70599 Stuttgart, Germany; 2 Central Institute for Decision Support Systems in Crop Protection (ZEPP), 55545 Bad Kreuznach, Germany

## Abstract

Wheat productivity is threatened by global climate change. In several parts of NW Europe it will get warmer and dryer during the main crop growing period. The resulting likely lower realized on-farm crop yields must be kept by breeding for resistance against already existing and emerging diseases among other measures. Multi-disease resistance will get especially crucial. In this review, we focus on disease resistance breeding approaches in wheat, especially related to rust diseases and Fusarium head blight, because simulation studies of potential future disease risk have shown that these diseases will be increasingly relevant in the future. The long-term changes in disease occurrence must inevitably lead to adjustments of future resistance breeding strategies, whereby stability and durability of disease resistance under heat and water stress will be important in the future. In general, it would be important to focus on non-temperature sensitive resistance genes/QTLs. To conclude, research on the effects of heat and drought stress on disease resistance reactions must be given special attention in the future.

## Introduction

Wheat (*Triticum aestivum* ssp. *aestivum*, *T. turgidum* ssp. *durum*) greatly contributes to global food security. However, wheat productivity is already threatened by global climate change (Porter et al. 2019), thereby countervailing some of the yield gains from breeding and other technological advances (Lobell et al. 2011) despite a CO_2_-fertilizing effect.

**Fig. 1:**
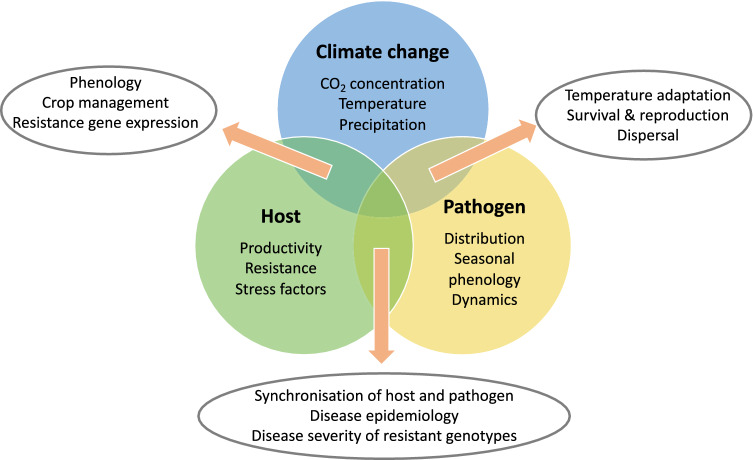
The ‘disease triangle’ concept simplified with focus on potential climate change effects. Few examples of driving factors are highlighted. Some likely consequences of future interactions are shown in the outside positioned circles. Other environmental parameters (e.g., soil type) and management options (e.g., fertilizer and irrigation inputs, soil tillage and sowing methods) are not shown, although they also influence the effects of future climate change on plant-pathogen interactions

Globally, the mean annual temperature increased since 1881 by about 1.0 °C (IPCC 2018). In Germany, the increase was about 1.5 °C, whereby warming was particularly pronounced in all years of the twenty-first century except 2010 (DWD 2021). Also, globally, the years of the twenty-first century were particularly warm (IPCC 2018). Climate change projections suggest that this trend will continue, although the magnitude will dependent on future CO_2_ and other greenhouse gas emissions, whereby successful reduction efforts will mitigate the trend of warming. For Europe, climate change projections (Jacob et al. 2014) suggest that until the end of the century the mean annual air temperature might increase in the range of 1.0–4.5 °C (RCP4.5 emission scenario) or 2.5–5.5 °C (RCP8.5 emission scenario) compared to 1971–2000. A temperature increase of up to 2.0 °C is possible until the middle of this century (Ceglar et al. 2019). Compared to 1971–2000, annual mean precipitation will be lower in southern Europe, to remain similar in central Europe, and to be higher in northern Europe (see Fig. 1 for more details in Jacob et al. 2014). In general, in most southern and central parts of Europe it will be dryer and warmer during the main crop growing period including spring and summer. This will increase the risk of extreme weather events, such as heat and drought periods.

Although crop disease problems will not generally increase till the end of this century (Juroszek and von Tiedemann 2015) crop productivity must be especially safe-guarded by increasing the input into the breeding goal ‘yield stability’ (Miedaner 2018). Breeding for resistance, preferably multi-disease or broad-spectrum resistance (Li et al. 2020; Miedaner et al. 2020a), against already existing and emerging diseases is an important component among others (e.g., water use efficiency, nitrogen use efficiency, low risk of lodging) to achieve yield stability (Chapman et al. 2012). Therefore, plant breeding must address both abiotic (e.g., heat, drought, waterlogging, salinity) and biotic stresses (Ceccarelli et al. 2010) including, for example, insect pests and pathogens, such as bacteria, phytoplasmas, viruses, fungi, oomycetes, and nematodes. The use of resistant or tolerant cultivars is cheap, environmentally sound and effective, unless pathogens quickly overcome the resistance (Juroszek and von Tiedemann 2011). Consequently, environmental stability and durability of resistance is crucial.

Herein, we will consider some effects in more depth which can be caused by climate change and illustrate them using three case studies of fungal diseases: (1) adaptation of pathogen populations to warmer conditions resulting in a higher fitness of the better adapted subpopulation/race (yellow rust), (2) re-emergence of old foes due to increasing temperatures (stem rust), and (3) change of the species’ assemblage and produced mycotoxins in a disease complex (Fusarium head blight, FHB). The resulting possible influences on disease breeding approaches in wheat are highlighted and discussed. We focus on NW Europe including Denmark, southern Sweden and Norway, Germany, Belgium, The Netherlands, Luxemburg, northern France, Ireland, United Kingdom, Switzerland, Austria, Czech Republic, Slovakia, and western Poland. However, most of our statements are valid also for other parts of Europe (e.g., Hungary, Romania, etc.) and elsewhere. Information related to the possible effects of enhanced CO_2_ on plant diseases can be found elsewhere (e.g., Manning and von Tiedemann 1995; Chakraborty and Datta 2003; Eastburn et al. 2011; Gullino et al. 2018).

## Impact of climate change on plant–pathogen interactions

For a disease to occur, a compatible pathogen and a susceptible host must interact, whereby the environmental conditions must be conducive for the disease (Fig. 1). The factor climate change in this disease triangle concept must be broadly seen, because it includes in addition to the long-term climatic conditions also other environmental factors, for example, soil type, below- and above-ground biome, and short-term prevailing extreme weather conditions, because they also greatly influence the plant-pathogen interactions. Fortunately, the farmer can influence this interaction, for example, by using a disease resistant cultivar, if available, or by strengthening the host plant fitness using appropriate fertilizer and irrigation inputs. For example, an application of 30 kg of K_2_O reduced spot blotch of wheat caused by *Cochliobolus sativus* similarly effective like one fungicide application (Duveillier et al. 2007).

Climate change can directly and indirectly affect pathogens and the respective diseases, which has been recently reviewed, for example, by Juroszek et al. (2020). A shift in temperature and other climatic conditions, such as altered precipitation, may result in various changes related to wheat pathogens which in general include (1) geographical distribution (e.g., range expansion or retreat, and increased risk of pathogen invasion), (2) seasonal phenology (e.g., coincidence of pathogen life cycle events with host plant growth stages and/or natural antagonists/synergists), and (3) population dynamics (e.g., over-wintering and survival, infection efficiency, latency period duration, number of generations of polycyclic pathogens). This may finally result in altered disease incidence and severity at a given location.

In general, all important life cycle stages of fungal pathogens (survival, reproduction including altered importance of sexual vs. asexual processes, and dispersal) are more or less directly influenced by temperature, humidity, light quality/quantity, and wind. Pathogens are particularly sensitive to temperature. For example, incubation and latency periods will be shorter due to warming (till supra-optimal temperature conditions will be reached), resulting in more generations of polycyclic pathogens per growth period (Wojtowicz et al. 2017). However, this may be countered by a decrease in moisture availability for successful secondary infections of polycyclic pathogens. On the other hand, temperature-tolerant subpopulations may develop due to a changing climate or they do have a high capability to successfully migrate, invade and establish at new geographic locations which are already facing warmer conditions (Chakraborty 2013).

Indirect effects are mediated through host-plant physiology and/or climate change-driven crop management adaptations, such as the introduction of irrigation, abolishment of soil turning operations to realize conservation agriculture, and shifted sowing dates, for example, due to accelerated crop development. Warmer mean air temperatures in Germany, especially in early spring since the end of the 1980s, have led already to the advancement of phenological phases of field crops, such as earlier beginning of stem elongation and flowering (Chmielewski et al. 2004). Fortunately, the timing of heading and flowering can be manipulated by farmers to a certain degree by adjusting sowing dates and/or using cultivars with different ripening patterns (early *vs.* late). Consequently, breeders must continue delivering appropriate and adapted wheat cultivars, assisting farmers to manage climate change-driven changes, be they direct or indirect.

Environmental conditions can affect disease resistance (Duveiller et al. 2007), whereby the host plant may have been weakened and/or the pathogen may have been strengthened or the function of a particular resistance gene could be influenced. For example, resistance to a disease can be temperature- and light intensity dependent (Carson and Vandyke 1994; Chakraborty et al. 2011). Also, water stress might influence the expression of resistance (Legreve and Duveiller 2010; Bidzinski et al. 2016). Unfortunately, there is not much specific and in-depth knowledge available on this important aspect. One of the first questions might be if the type of resistance can be important? For qualitative resistance, presumably, only a small number of genes do have a temperature-dependent function (see below), because usually expression of effective major genes is quite stable across environments. However, research just started to look in-depth on a molecular basis at this aspect. For quantitative resistance, it is well established that the ranking of genotypes might greatly change due to site-specific environmental effects. Thus, it appears to be more likely that drought and heat stress influence its expression. However, it can be assumed that abiotic stress does not completely counters quantitative resistance, but ‘just’ decreases the degree of resistance. The background is that usually many minor genes are contributing to quantitative resistance and it is very unlikely that all minor genes (or QTLs) are similarly temperature sensitive, although there is a lack of research to support this assumption. One reason might be that such quantitative effects caused by temperature or drought are difficult to prove experimentally, in contrast to a qualitative reaction (yes or no), especially under field conditions, because the abiotic stress conditions may not only affect resistance mechanisms, but also the general host physiology and pathogen growth and aggressiveness within the plant.

## Change of the relative importance of fungal diseases

In NW Europe, wheat is economically affected by about a dozen of fungal diseases (Table 1). Basically, warmer temperatures favor most fungal species up to their individual upper temperature threshold (Racca et al. 2011). However, the water availability might be a limitation in future. Some pathogens, such as the rain-splashed fungus *Zymoseptoria tritici*, are dependent on several and considerable rainfall events and a correspondingly long dew phase throughout the vegetative growing period of wheat during springtime, especially to finally reach and infect the upper yield-relevant leaves. In contrast, others such as many *Fusarium* species may only need a single precipitation event of about 2–3 mm during flowering, subsequently causing severe Fusarium head blight (FHB) including mycotoxin contamination on susceptible cultivars. Few others need only overnight dew, such as *Puccinia triticina*, subsequently causing leaf rust. Consequently, Septoria tritici blotch (STB) caused by *Z. tritici* might become in the future mainly critical at the end of the (warmer) winter and early springtime, while during late springtime and early summer sufficient rainfall and dew might get a limiting factor for this disease. On the other hand, most likely, FHB of wheat and the wheat rusts will profit more from global warming in NW Europe than STB, because they are less dependent on frequent and relative high rainfall events, latter projected to be less frequent in the future. These kinds of ‘speculations’ based on expert knowledge related to the future importance of wheat diseases (reviewed by Juroszek and von Tiedemann 2013a) are in agreement with most simulation studies, where a wheat disease model is driven by climate change scenarios to estimate the future disease risk based on mathematical equations (Table 1).

**Table 1:** Simulated fungal disease risks of winter wheat in Europe using plant disease models driven by climate change scenarios, usually downscaled to a regional level. Projections until 2050 considered

Disease (Pathogen)	Country (Region)	Change of disease risk^a^	Reference
Powdery mildew (*Blumeria graminis*)	Germany (NRW) Germany (LS)	+ −	Volk et al. (2010) Racca et al. (2012)
Leaf rust (*Puccinia triticina*)	Germany (NRW) Germany (LS)	+ +	Volk et al. (2010) Racca et al. (2012)
	Europe	+	Bregaglio et al. ( 2013)
	Luxemburg	+	Junk et al. (2016)
	Scotland Poland France France	+ + + +	Davies et al. (2007) Wojtowicz et al. (2017) Caubel et al. (2017) Launay et al. (2020)
Yellow rust (*Puccinia striiformis*)	Germany (NRW) Europe	+ +	Volk et al. (2010) Bregaglio et al. (2013)
Stem rust (*Puccinia graminis*)	NW Europe	+	Prank et al. ( 2019)
	NW Europe, UK	+	Davies et al. (2007)
Eyespot (*Oculimacula yallundae*)	Germany (NRW)	o	Volk et al. ( 2010)
Septoria tritici blotch (*Zymoseptoria tritici*)	Germany (NRW) France	+ -	Volk et al. (2010) Gouache et al. (2013)
Septoria nodorum blotch (*Parastagonospora nodorum*)	Germany (NRW)	o	Volk et al. (2010)
Tan spot (*Pyrenophora tritici-repentis*)	Germany (NRW) Germany (LS)	o +	Volk et al. (2010) Racca et al. (2012)
Fusarium head blight (*Fusarium* spp.)	Germany (NRW)	+	Volk et al. (2010)
	Scotland	+	Davies et al. (2007)
	UK	+	Madgwick et al. (2011)

^a^Change of disease risk: − decrease, o unchanged, + increase, NRW = Northrhine-Westfalia, LS = Lower Saxony

**Footnote 1**: Most studies consider the infection risk (e.g., Volk et al. 2010; Racca et al. 2012; Bregaglio et al. 2013; Junk et al. 2016; Caubel et al. 2017; Launay et al. 2020), whereas few studies consider inoculum accumulation risk (Volk et al. 2010) or wind velocity patterns supporting long-distance spore distribution (Prank et al. 2019) or duration of latency period (e.g., Wojtowicz et al. 2017) or disease incidence (Madgwick et al. 2011) or disease severity (Gouache et al. 2013). **Footnote 2**: Speculations based on expert knowledge usually consider the complete disease cycle (e.g., Boland et al. 2004). These are not shown in Table 1, but can be found in the review article by Juroszek and von Tiedemann (2013a) related to future risks of wheat diseases. **Footnote 3**: Results of simulations of future disease risk until 2100 are not shown; however, usually the risk continues to increase (e.g., Racca et al. 2012; Junk et al. 2016; Caubel et al. 2017; Wojtowicz et al. 2017; Launay et al. 2020) or to decrease (e.g., Gouache et al. 2013), respectively. Rarely, the risk of a certain disease first increased (2050) and subsequently decreased (2100) in the simulation studies. For review see Juroszek and von Tiedemann (2013a, b, 2015)

However, the outcomes of the disease risk simulation studies are dependent on many factors including, for example, (1) the specific plant disease model used, (2) the specific climate change scenario used, and (3) the specific downscaling method used. Last but not least, it is also important which specific location(s) were considered in the model runs (e.g., lowlands vs. mountains, north vs. south), although located in the same country (Gouache et al. 2013; Launay et al. 2020). Anyhow, the same is true for breeding goals and requirements, which usually must also be fine-tuned and adapted to the specific cultivation location in the same country. Therefore, it is not surprising that STB is projected to increase in Germany (Northrhine-Westfalia, NRW), whereas the same disease is projected to decrease in France (Table 1) or that wheat powdery mildew is projected to increase in NRW, whereas it is projected to decrease in Lower Saxony, both German Federal States (Table 1). For the diseases of eyespot caused by *Oculimacula yallundae*, Septoria nodorum blotch (SNB) caused by *Parastagonospora nodorum*, and tan spot caused by *Pyrenophora tritici-repentis*, disease infection risk will not change in NRW compared to the baseline period, while for Lower Saxony a higher infection risk for tan spot is projected (Table 1). SNB might become important in southern Scandinavia according to our speculation, although we could not find respective simulation studies. However, these results are based on different material and methods. It is, therefore, risky for plant breeders to draw conclusions on just a few simulation studies related to a specific plant disease.

Table 1 suggests that the risk of most wheat diseases will increase until 2050 in Europe. In particular, there are many studies on wheat leaf rust available and all of them simulate an increasing risk of wheat leaf rust, independently of the disease cycle parameter simulated, namely inoculum accumulation during winter- and early spring-time (Volk et al. 2010), infection risk (e.g., Racca et al. 2012; Bregaglio et al. 2013; Junk et al. 2016; Caubel et al. 2017; Launay et al. 2020), and duration of latency period (Wojtowicz et al. 2017), presumably resulting in increased incidence and severity of wheat leaf rust this century in NW Europe.

However, in general, caution is needed, because input data of past and currently used plant disease models may be outdated. Presumably, the simulations for yellow rust cited in Table 1 are not up-to-date anymore, because the disease simulation models were run with the data of the ‘old’ European races of *P. striiformis.* However, meanwhile the Warrior, Warrior (-) races and their descendants are prevalent in NW European wheat fields (GRRC 2020) and they show a different response to temperature (see below). Therefore, several German authorities (e.g., ZEPP, JKI, and the Plant Protection Services of several German Federal States) have started in 2020 a comprehensive joint project (ValiProg) with the goal to evaluate and, if needed, to up-date important disease forecasting models of arable crops including wheat. This might alter the simulations of future disease risks in cases where pathogens have adapted to changed environmental conditions. Therefore, we have restricted the results shown in Table 1 to the middle of this century (2050), although results until 2100 are available (see Footnote 3 below Table 1).

Unfortunately, not a single simulation study of future disease risk in wheat (or any other crop) considered the disease resistance status of the cultivar(s) used in the model runs (reviewed by Juroszek and von Tiedemann 2015). Nevertheless, one study (Gouache et al. 2011) considered the cultivar choice of wheat related to the harvest date (early vs. late maturing cultivar) to escape severe disease impact at the end of the wheat growing period. Racca et al. (2013) considered the wheat sowing date in the model runs, also to avoid severe disease development, whereby the beginning of the disease cycle was targeted (usually the later the sowing date of winter wheat, the lower the initial disease infection risk). However, in general, the disease risk simulation studies rarely included an adaptation option (e.g., timing of sowing, choice of cultivar), although this approach is particularly valuable, because it informs about future possibilities to minimize disease risk (Juroszek and von Tiedemann 2015).

Wheat leaf rust is simulated to be favored with high confidence (in total 8 simulations) by climate change. Resistance breeding to leaf rust, however**,** is very demanding, because the monogenically inherited all-stage (seedling) resistance genes are notorious to get ineffective, often within a short time period, due to a usually rapid development of virulent races of *P. triticina*. Although race-specific resistance genes *Lr9, Lr19,* and *Lr24* are still effective in Europe (Serfling et al. 2013), newly registered cultivars quite quickly become susceptible to leaf rust (Laidig et al. 2020). Thus, race-nonspecific genes conferring adult-plant resistance (APR), for example *Lr34, Lr46, Lr67,* or *Lr68*, have been proposed to be used for practical breeding (Singh et al. 2011b; Spielmeyer et al. 2013). These genes, except *Lr68*, are also effective against multiple other pathogens (see below ‘Opportunities in Resistance Breeding Strategies’) and have been proven to be durable. The *Lr34* gene, for example, provides partial resistance to all known leaf rust races and is effective since almost 100 years (Ellis et al. 2014). Because the effect on leaf-rust severity in NW Europe, however, is moderate at best, *Lr34* should be pyramided with other APR genes or with still effective all-stage resistance genes to allow for a higher effect and durability. Also, *Sr2/Yr30* closely linked to *Lr27* belongs to this class of pleiotropic, partial APR genes. Combinations of 4–5 of such genes usually result in a high level of resistance. In improved CIMMYT germplasm, a high diversity for adult-plant resistance occurs for all three rusts; however, only relatively few genes are characterized in detail (Singh et al. 2011b). In a study from Uruguay, *Lr68* alone reduced AUDPC for leaf rust by 51%, together with *Lr34* and *Sr2* even a reduction of 73% was observed (Silva et al. 2015). Therefore, a combination of several different complementary functioning *Lr* genes is one strategy to improve leaf rust resistance in wheat, and another would be to implement quantitative resistance sources. For example, 144 unique QTL were reported that resulted in 35 meta-QTL when projected on the wheat reference map (Soriano and Royo 2015).

According to the simulations (Table 1), also FHB (in total 3 simulations = ‘medium confidence’) is projected to be favored by climate change due to the thermophilic nature of the respective *Fusarium* species, while they are moderate in moisture requirements. Presumably, this is also true for *P. graminis* causing wheat stem rust and *P. striiformis* causing wheat yellow rust (in total 2 simulations each = ‘some confidence’). Warming will not only favor the above-ground leaf pathogens but also the prevalence and growth of economically important soil-borne plant fungal pathogens, such as *Alternaria*, *Fusarium* and *Phoma* species (Delgado-Baquerizo et al. 2020). In addition, most insect pest species might thrive in NW Europe due to warming (Deutsch et al. 2018), among them vectors of plant pathogens, such as viruses and phytoplasmas (Ordon et al. 2009) and insects that feed on plants (Miedaner 2018), thereby creating gates for pathogen entry, for example, for *Fusarium* species which produce mycotoxins.

## Case study 1—yellow rust as an example of a highly temperature-adaptive pathogen

**Fig. 2:**
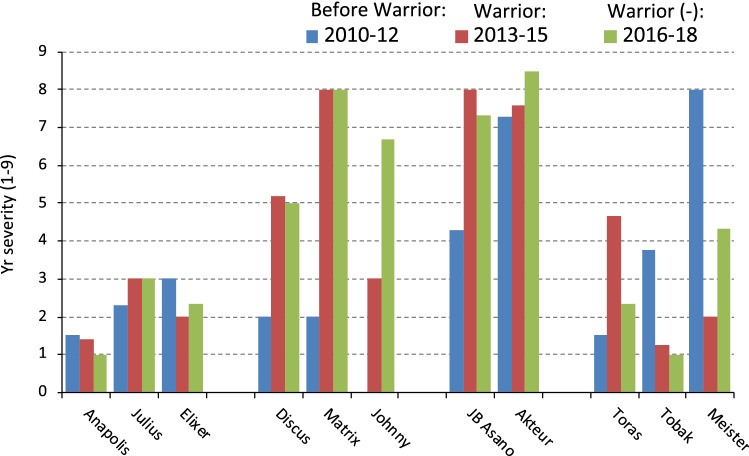
Yellow rust severity of selected German cultivars in the adult-plant stage (EC 49–71) inoculated in the field with mixtures of *Yr* races that were predominant in the respective year; *Yr* severity was tested in the given years at Berlin-Dahlem and based on the 1–9 scale, with 1–3 = resistant, 4–6 = intermediate, 7–9 = susceptible (Kerstin Flath, Julius-Kuehn Institute, pers. commun.)

Before 2011, yellow rust was a disease of maritime climate zones caused by a typical NW‐European population (Hovmøller et al. 2016, now named *PstS0*) and epidemics occurred every 5–10 years. When the new races, Warrior and Kranich (now *PstS7* and *PstS8*, resp.), arrived in Europe in 2011, the yellow rust disease patterns in the fields totally changed. The new races were detected on both wheat and triticale in many European countries, had more virulences than the old strains, were more aggressive, especially on adult plants of wheat and produced more telia on infected leaves (Hovmøller et al. 2016). Subsequently, large epidemics occurred in 2013 and 2014 and also in several following years due to their high frequency in NW Europe. In the meantime, descendants of Warrior, like Warrior (-) and others (now *PstS10*), appeared (GRRC 2020). The new races resulted in considerable changes in the resistance level of many cultivars (Fig. 2). Some cultivars (e.g., Anapolis) remained resistant; however, several formerly resistant cultivars (e.g., Matrix) with ratings between 2 and 3, became susceptible with ratings between 7 and 9. Already susceptible cultivars (e.g., Akteur) became even more susceptible due to the higher aggressiveness of the new races. This example clearly shows that it is crucial to regularly monitor rust populations across years in order to timely guide resistance breeding approaches; otherwise reactions of breeders to changing conditions are too late.

Without looking at complex interactions, there is a clear tendency for the new yellow rust races to infect at warmer temperatures. The Warrior race infected susceptible wheat cultivars also in areas that have not been regularly attacked by the ‘old’ NW European strains, like southern Germany, Austria, northern Italy (Po valley) and even Spain (GRRC 2020). Indeed, Warrior and Warrior (-) showed on a susceptible cultivar at 26 °C/10 °C disease severities up to 12% in seedling stage while an old European race had its optimum at 20 °C/10 °C and was not able at all to infect at the higher temperature regime (Kabakeris et al. 2020). Differences in temperature adaptation were also reported for isolates originating from northern and southern France with a particular advantage of the southern isolates in the warmer Mediterranean climate (Mboup et al. 2012; Vallavielle-Pope et al. 2018).

Already in the year 2000, two other isolates of yellow rust with a high temperature adaptation were firstly detected, *PstS1* and *PstS2* (Walter et al. 2016). They brought the disease to geographical regions that were believed to be too warm for severe yellow rust epidemics, Western Australia and the South Eastern USA (Milus et al. 2009). Before their emergence, in both regions, yellow rust rarely exceeded trace levels of disease. The new races sporulated three days earlier (faster) and produced 370% more spores (area/day) at high temperatures (28/12 °C) than the ‘old’ races (Milus et al. 2009). Molecular studies revealed that both strains originated from East Africa, *PstS1* as early as in 1980s and then spread to the Americas in 2000 and to Australia in 2002 (Walter et al. 2016).** Pst**S2 which evolved from *PstS1* became widespread in the Middle East and Central Asia and was firstly detected in Europe in 2000, but became not prevalent here till today (GRRC 2020).

Wheat rust diseases can be controlled by fungicides, monogenic all-stage (seedling) resistances, monogenic or quantitative adult-plant resistances. The use of winter wheat cultivars resistant to yellow rust is quite common in Germany. About 75% of the cultivars included in the Descriptive List of Varieties (BSA 2020) are resistant to yellow rust with a score ranging from 1 to 3. Information on the genetic basis of these resistances is not available; however, an association study considering about 150 German winter wheat cultivars indicated a quantitative inheritance with 13 QTLs detected across three locations, each explaining 1.3–10.8% of the genotypic variance in the adult-plant stage (Miedaner et al. 2020a). The allelic effects of the main QTLs showed that 71–87% of the winter wheat lines already carried the resistance alleles. Seedling tests revealed that *Yr5, Yr15*, and *Yr24* still have only low (< 5%) virulence frequencies in Germany (Flath, pers. commun.). The cultivar ‘Julius’ has presumably a quantitative adult-plant resistance, because it is still resistant in the field when older (see Fig. 1, assessed at EC40-50), while it is susceptible in seedling stage to the majority of actual isolates (Kerstin Flath, Julius-Kuehn Institute, pers. commun.). Today, however, ‘Julius’ is only moderately resistant any more (score 5 on the 1–9 scale, BSA 2020). Thus, there is some evidence that quantitative adult-plant resistance sources against yellow rust are available in commercially used wheat cultivars in Germany, although individual major genes might be also involved. Some evidence for this hypothesis is provided by the fact that, fortunately, the new aggressive Warrior and Warrior (-) races of *P. striiformis* have not over-come all yellow rust resistant wheat cultivars (Fig. 1). However, comprehensive inheritance studies are necessary to support this hypothesis.

## Case study 2—stem rust as an example of a re-emerging foe

Wheat stem rust is a devastating disease that is one of the main causes of yield loss in Africa, especially since the occurrence of the highly aggressive Ug99 race and lineages derived therefrom (Singh et al. 2011a). In Europe, the disease has not been epidemic since the 1950s (Bhattacharya 2017). One reason might be that farmers switched from spring to winter wheat cultivation. However, in 2013 stem rust re-occurred in Central Germany causing a small regional epidemic and it also appeared in this year sporadically in the UK and Denmark (Table 2).

**Table 2:** New epidemics of wheat stem rust in Eurasia (Shamanin et al. 2016; Saunders et al. 2019; https://rusttracker.cimmyt.org/?p=7083)

Year	Region	Damage
2013	Central Germany UK, Denmark	Regional epidemic, winter wheat sporadic occurrence
2015(+ 2016)	Western Siberia/Russia and Kasachstan	> 1 million hectares spring wheat, 20–30% yield loss
2016	Sicily/Italy	~ 20–30.000 ha durum wheat, 20–60% disease incidence
2017	Central Sweden	Late-maturing wheat and barley

In Europe, during the past few years the largest stem rust outbreak occurred in southern Italy, Sicily in 2016 where durum wheat was severely infected on several thousands of hectares. One year later, in summer 2017, an outbreak occurred far north in Central Sweden, presumably originating from barberry (*Berberis vulgaris* L.), the sexual host plant of *P. graminis* (Berlin 2017). Also, in Germany (Flath et al. 2020) and the UK (Lewis et al. 2018), wheat stem rust was frequently isolated from wild barberry leaves, showing that the fungus is permanently prevalent and can successfully finish its whole life cycle in NW Europe. As stem rust can be readily field inoculated on winter wheat in Germany (Flath et al. 2018) and Austria (Oberforster et al. 2010), there must be climatic and/or epidemiological reasons why *P. graminis* does not regularly cause stem rust epidemics in wheat crops throughout Europe.

**Fig. 3:**
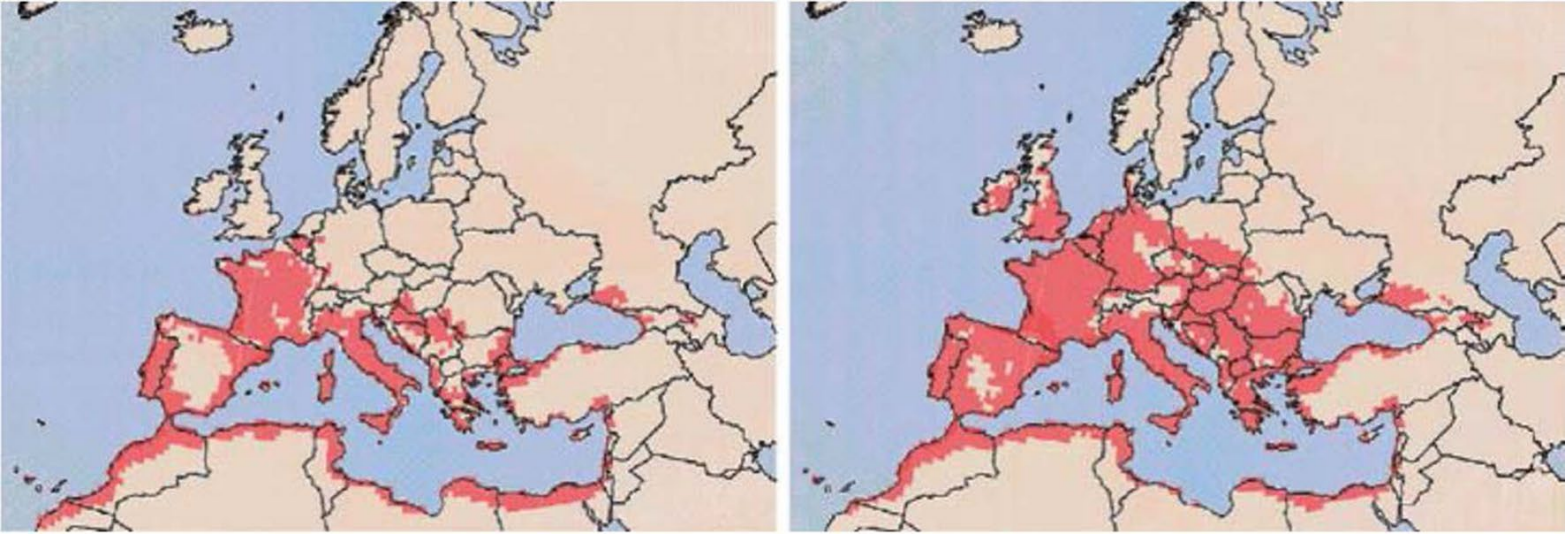
Stem rust risk in Europe under average climate conditions for the last 30 years (left) and predicted climate in 2050 (right) (Davies et al. 2007, with permission of the authors)

Davies et al. (2007) reported that wheat stem rust is a potential re-occurring threat for NW Europe, for example, in 2050 (Fig. 3). These authors used the current climatic conditions of the wheat production area in Uganda/Africa and compared them with the European climate change scenarios for 2050. According to this climate matching approach, *P. graminis* will potentially be able to cause stem rust epidemics in most countries of NW Europe including Ireland and southern England.

In addition, Prank et al. (2019) simulated for the end of the twenty-first century an increase of about 40% of urediniospore emitting potential of stem rust, potentially affecting wheat crops, due to changing future wind velocity patterns. Due to warming, also an expansion of regions in Europe (including Germany and eastern Europe) is possible where stem rust can overwinter in uredinial stage. Lewis et al. (2018) assume that although the risk of spore germination and appressorium formation may increase, the wet conditions required for leaf penetration are unlikely to become more common in the future. However, because sexual recombination of stem rust via the host plant barberry is possible in NW Europe, stem rust might adapt to new climatic conditions similarly successful like yellow rust.

Stem rust resistance is an important goal of international wheat breeding networks, whereby CIMMYT plays a key role. In NW Europe, however, stem rust resistance was neglected in wheat breeding programs due to the absence of epidemics in NW Europe during the past 50–60 years. However, since 2013 wheat stem rust resistance is again on the agenda of breeders targeting the European market including Germany. Out of 79 German winter wheat cultivars tested in seedling and adult-plant stage (field) 37% carried *Sr38*, 14% *Sr31* and 10% *Sr24* (Flath et al. 2018). The latter two *Sr* genes fully protected winter wheat cultivars from stem rust at the adult-plant stage in the field. For further nine cultivars with high susceptibility in seedling stage, quantitative adult-plant resistance was suggested. In a newer study of the BMEL-sponsored project ‘GetreideProtekt,’ only 9% of the tested 280 European cultivars were field resistant (< 4% severity) to stem rust in the adult-plant stage (Miedaner and Flath, unpublished data). In the UK, 20% of currently grown winter wheat cultivars were estimated to be resistant to the first modern stem rust isolate UK-01 detected in 2013 (Lewis et al. 2018). On a world-wide basis, CIMMYT promotes the use of *Sr2*, a gene with partial resistance to stem rust of high durability that should, however, be used in conjunction with other resistance genes to protect wheat cultivars effectively (Ellis et al. 2014). In general, as many new resistance sources as possible should be detected and if useful be introduced in future wheat cultivars preferable in resistance gene pyramids. As yellow rust and stem rust resistances are not correlated, although some seedling resistance genes might confer both (see multi-disease resistance), stem rust resistance programs should be initiated in public and private wheat breeding organizations in NW Europe to cope with the increasing threat, namely the re-emergence of the old foe *P. graminis*.

## Case study 3—shift in *Fusarium* species and related mycotoxins

Mycotoxins are a worldwide threat to the security of food and feed. *Fusarium* species causing FHB of wheat produce mainly three groups of mycotoxins: trichothecenes, the most important are deoxynivalenol (DON), nivalenol (NIV), and T2-/HT-2 toxin, zearalenone (ZON), and fumonisins (FUM). They have been found in EU wheat grain in 61, 14, 21, 30, and 79% of 45.000 samples analyzed, respectively (Gareis and Zimmermann 2003). Of course, this is only a snapshot of a probable mycotoxin contamination situation at a certain time point, because *Fusarium* species are shifting constantly and consequently also their mycotoxin production and wheat grain contamination is changing. The responsible *Fusarium* species have in vitro a wide range of optimal temperatures (Paterson and Lima 2011), whereby the trichothecene production is favored by moderately warm temperatures (optimal 15–25 °C, upper threshold 32 °C) and high humidity, while FUM is produced mainly under relative warm weather conditions (optimal 24–30 °C).

Fusarium diseases are caused by combinations of different species. For FHB in wheat up to 20 different *Fusarium* species are co-existing (Timmusk et al. 2020). The most common species are *Fusarium graminearum* (*Gibberella zeae*), *F. culmorum*, *F. avenaceum* (*G. avenacea*), *F. poae*. They have different toxin production profiles, while *F. graminearum* and *F. culmorum* mainly produce DON or NIV, and ZON, *F. avenaceum* produces the non-trichothecenes moniliformin (MON) and beauvericin (BEA), and *F. poae* mainly NIV and T-2/HT-2 toxins (Venkatesh and Keller 2019). It should be noted, that these mycotoxins have different chemical structures, properties, and toxic and harmful effects on humans and livestock.

The *Fusarium* species have different ecological adaptations and environmental preferences what might affect their relative occurrence within the *Fusarium* complex. *Fusarium avenaceum* is favored by cool and moist conditions at the end of summer season (Parikka et al. 2012). Also *F. culmorum* is adapted to cool temperatures, because this species was in the 1980s and early 1990s mainly found in the UK, The Netherlands, northern Germany, and northern Poland. However, the frequency of isolations of *F. culmorum* sharply decreased in the last decades in these regions, whereas *F. graminearum* increased (Waalwijk et al. 2003). Because both species have a similar mycotoxin profile, presumably, this did not significantly affect contamination profile of wheat grains; however, a drift towards the other mentioned species would sharply change the mycotoxin profiles in the cereal grains, for example, to more FUM in grains. However, in several European countries a high percentage of isolations of *F. poae* was reported with a declining frequency of *F. graminearum* in the last years (Valverde-Bogantes et al. 2019). This was explained by higher temperatures and drier conditions during wheat anthesis. Accordingly, investigations on *Fusarium* biodiversity in wheat show an astonishingly high percentage of isolations from *F. poae* in some regions of NW Europe (reviewed by Becher et al. 2013). Interestingly, *F. poae* symptoms are rarely observed in the field, consequently the fungus reduces yield less than *F. graminearum*. However, *F. poae* produces mainly NIV and T-2/HT-2 toxins that both have a higher toxicity to humans and livestock than DON. Thus, a replacement of *F. graminearum* by *F. poae* would worsen the implications for food and feed security, although, presumably, grain yield decline of wheat would be lower.

Considering *F. graminearum*, still the worldwide most common species in the FHB complex, warm weather is detrimental for food and feed security, because more DON can be produced, at least till a maximum of 32 °C (Paterson and Lima 2011). In addition to warming, future mycotoxin load of wheat grain will be affected by future moisture conditions including rainfall, relative humidity, duration of ear wetness, and water activity in grain. However, not much is known on the minimum moisture requirements for each of the *Fusarium* species. Therefore, strictly seen, simulations and predictions based on moisture-related parameters are less reliable compared to those which are based on temperature-related parameters.

Nevertheless, a simulation study on future DON content in wheat in 2050, based on hourly data of temperature, relative humidity and rainfall from The Netherlands, Norway, Sweden, and Finland revealed that DON contamination will increase on average by the factor 1.36 in the most likely scenario (van der Fels-Klerx et al. 2012). However, in a follow-up paper the authors stressed the fact that even for a small country like The Netherlands, the models show a large variation among regions with a slight decrease in one region and an increase in another region (van der Fels-Klerx et al. 2013), presumably reflecting variable small-scale precipitation and air humidity patterns among other reasons.

A northward expansion of FHB disease in wheat can be expected. In Scotland, for example, infection level is at present on a low level, if FHB is occurring there at all. However, simulations predict that there will be a greater risk in the future throughout the east coast, especially because considerable maize cultivation might be introduced in Scotland till 2050 (Davies et al. 2007). Although Scotland will have the greatest relative increase in the UK, the highest absolute incidence of FHB till 2050 is simulated for central southern England with, on average, 10–16% of wheat plants affected by the disease in a particular field (Madgewick et al. 2011). In northern Europe, milder temperatures and more rainfall during late summer are predicted, presumably leading to an increased incidence of *Fusarium* infection in wheat (Parikka et al. 2012; Moretti et al. 2019). To summarize, given the high plasticity of *Fusarium* species like *F. culmorum* (Castiblanco et al. 2020) and presumably others of the 19 *Fusarium* species in this disease complex, their continuous adaptation to changing weather conditions must be expected. Therefore, FHB in wheat will remain an important aspect in disease resistance breeding approaches, because if one individual *Fusarium* species is performing poor, another one will most likely perform well (Juroszek and von Tiedemann 2013b). They can replace each other, whereby mycotoxin type and level will be dependent on which *Fusarium* species will dominate in a particular year or region, mainly controlled by the prevailing weather conditions, although a few species, such as *F. graminearum*, appear to be dominant across years.

Intensive genetic research during the last three decades revealed a few QTLs with major effects for FHB resistance of Chinese origin (e.g., *Fhb1, Fhb2, Fhb4,* and *Fhb5*) which have been transferred into wheat breeding across the globe (Miedaner and Korzun 2012; Ma et al. 2020) and many minor-effect QTLs reported in durum wheat (Prat et al. 2014) and bread wheat (Venske et al. 2019). In the latter study, about 550 FHB QTLs were found across the entire genome (i.e., A, B, D subgenomes) and have been reduced to 65 meta-QTL. Most of the QTLs contributed only small proportions of the genotypic variance (for review see Buerstmayr et al. 2020). In Europe, mainly recurrent selection approaches for QTLs of native origin revealed cultivars with high to moderate FHB resistance. The main challenge today is to combine these QTLs for FHB resistance with superior agronomic performance, especially straw shortness, earliness, grain yield, and quality.

## Opportunities in disease resistance breeding strategies

For both commercial and public breeders, it is critical to decide which disease resistance trait should be implemented in the breeding process, because each additional trait reduces the selection intensity for all other traits, such as yield potential and yield stability, usually leading to more expensive and longer breeding process (Miedaner and Korzun 2012). However, time is a crucial aspect, not just because of higher investment, but also due to presently accelerated climate change conditions. Often, the decision of a breeder is influenced, for example, by the yield loss potential of a particular disease in a certain region, the preferences of the respective farmers, the availability of resistance sources including their likely durability which is also dependent on the prevailing virulent strains within a pathogen population of the respective region.

Resistance breeding is at present practiced for most wheat diseases mentioned above, although in different intensities. Gain from selection between 1983 and 2019 was highest for resistances to powdery mildew, leaf rust, SNB, and STB, but rather low for yellow rust resistance (Laidig et al. 2020). In future, the focus should be especially on resistances managing the three wheat rust diseases and FHB, what does not mean that other wheat diseases should be neglected. For STB, it might be appropriate to select resistance sources specially to manage STB during young plant developmental stages, because a high infection risk might be earlier in the season than today due to the future milder winter and early spring conditions. However, later in the season, STB disease risk might get lower than today due to predicted drier summer conditions likely impacting both rain-splashed distribution of *Z. tritici* spores to the upper leaves and infection risk if spores would, nevertheless, reach the upper leaves.

Future temperature conditions may support the use of high-temperature adult-plant (HTAP) resistance genes against yellow rust also in NW Europe. They are effective after stem elongation when average night temperatures remain above 10 °C and day temperatures are between 25 °C and 30 °C (Qayoum and Line 1985), although novel studies suggest that they are also effective at lower temperatures of about 18 °C and below, at least shown in the UK for the HTAP resistance gene *Yr36* (Segovia et al. 2014). In addition, Segovia et al. (2014) found that young wheat plants, but not seedlings, were also protected by this gene. Therefore, it is critical to re-evaluate older studies due to the occurrence of new races. Nevertheless, in the USA, HTAP resistance genes are widely used in wheat cultivars since the 1960s and are recommended for worldwide use (reviewed by Chen 2013). The level of resistance conferred by HTAP resistance sources is known to be moderate only and affected by temperature and humidity but has a high durability and is race nonspecific (Line and Chen 1995). Typically, *Yr18, Yr29, Yr34, Yr36, Yr39*, *Yr48, Yr52*, *Yrns-B1,* and at least 80 QTLs from 33 wheat cultivars are described as HTAP resistance (Chen 2013). Among the resistance sources are well known European cultivars (Cappelle Desprez, Récital/Camp Rèmy, Renan, Alcedo, Apache, Naxos). It should be evaluated if high-temperature adult plant resistance genes are useful to manage not only yellow rust in the future, but also other wheat rust diseases. In contrast, resistance sources could be selected that are not greatly influenced by temperature to broaden their range of use.

Bryant (2013) concluded that in-depth investigation of temperature sensitivity of resistance genes in wheat is long overdue, because molecular understanding of resistance gene(s) processes has advanced and new technologies and approaches are in place. Cohen and Leach (2020) using a combination of phenotypic and transcriptomic studies with molecular-genetic analysis in both the plant host and the pathogen, have already provided valuable mechanistic insights into plant responses to combined abiotic and biotic stresses, namely pathogen attack under high temperature. To realize a high stability and durability of disease resistances under heat and drought stress, a multi-environment selection under these conditions is necessary. Further, water stress on disease resistance has been neither investigated for specific *R* genes nor for quantitative resistances in wheat. However, at least a few more in-depth investigations related to the temperature sensitivity of disease resistance genes are available considering mainly yellow rust, whereby both High Temperature Seedling Plant Resistance (e.g., Wang et al. 2019, 15 vs. 20 °C tested) and High Temperature Adult Plant Resistance (e.g., Coram et al. 2008, see above) were considered. Leaf rust (e.g., Broers and Wallenburg 1989) and stem rust (e.g., Chen et al. 2018) were rarely investigated. Unfortunately, leaf rust resistance is far less investigated compared to yellow rust, although the risk of wheat leaf rust will particularly increase in the future in NW Europe (see Table 1) and elsewhere.

Butterworth et al. (2010) suggested that strategies for breeding cultivars with improved resistance to pathogens will need to include trials in countries with a warmer climate that represents predicted climates in yet cooler locations. This is in agreement with Challinor et al. (2016) who reported that current breeding programs are usually ‘one step behind’ being optimal concerning the future temperature conditions cultivars will face once released and adopted by farmers, because the breeding process is, relatively seen, too slow when considering the current accelerated global warming trend.

A future scenario should be to favor multi-disease resistances (MDR)*, i.e.,* genes or QTLs that confer resistance to several diseases. In wheat, a few monogenic APR genes are known that inherit partial resistance to several rusts, powdery mildew, and other traits: *Sr2/Yr30/Lr27*, *Lr34/Yr18/Sr57/Pm38, Lr46/Yr29/Sr58/Pm39,* and *Lr67/Yr46/Sr55/Pm46* (Singh et al. 2011b; Silva et al. 2015). In addition, *Lr34* and *Lr46* provide resistance to spot blotch caused by the hemibiotrophic fungus *Bipolaris sorokiniana* by *Sb1* and a minor QTL, respectively (Lillemo et al. 2013). Recently, it was shown that lines possessing both *Sr2* and *Lr34* significantly enhanced APR to all three rusts in field trials (Randhawa et al. 2018). Similarly, several QTL have been described in wheat that confer multiple resistances to a combination of two of the diseases STB, SNB, and FHB (Miedaner et al. 2012). In a more recent approach, nine MDR QTL have been detected in a wheat diversity panel for resistances to four diseases, powdery mildew, yellow rust, stem rust, and FHB that have not been shown up in the individual disease resistances (Miedaner et al. 2020a). Combining such MDR QTL by applying marker-assisted or genomic selection seems a promising approach and should allow to react faster to changing pathogen populations or changes in the importance of diseases. This is especially valid, because for all disease resistances many QTL have been described, mainly with only small additive effects and a high environmental sensitivity, making it very tedious to combine many of them in one cultivar. For traditional resistance breeding strategies that could be applied for any of these types of resistance please refer to Miedaner (2016).

For quantitative resistances inherited by many QTL with small effects, genomic selection (GS) seems to be a valuable tool. GS aims for capturing all additive genetic variance of a trait by using a large number of markers. This has been shown to be more accurate than marker-assisted selection approaches that capture only the effects of large effect QTLs in most studies (e.g., Rutkoski et al. 2012; Mirdita et al. 2015; Miedaner et al. 2019). The prediction ability of genomic selection was high amounting to 0.6 for FHB and 0.5 for STB resistances, similar values were observed for the rusts. Based on genome-wide marker data and representative training populations, genomically estimated breeding values can be used to select for resistant progenies that have only been genotyped. In an experimental approach, Herter et al. (2019) found a 10% improved FHB resistance when selecting only genomically based on a 15 k SNP assay. GS for disease resistance is especially promising, because many commercial breeding programs already apply it for selection of grain yield. The same marker data could also be used for selection for MDR when the respective training populations are additionally phenotyped for combined disease resistances. In programs with a higher impact on disease resistances, an indirect selection by genomic data could already be performed in the early stages of a breeding program with a training set from the previous years (Galiano-Carneiro et al. 2019). This can be expected to lead to a shift of the population mean towards higher resistance and thus to a higher probability for selecting superior MDR cultivars. Taken together, MDR wheat cultivars will play an important role to mitigate future increasing disease risks in NW Europe including wheat rusts and FHB.

## Conclusions

The review highlights several challenges for resistance breeding under the conditions of global climate change: (1) Increasing risk of diseases, such as leaf rust and FHB, (2) re-emergence of old enemies, such as stem rust, (3) highly adaptive pathogens, such as *P. striiformis*, (4) emergence of new yet unknown pathogens, (5) changes in *Fusarium* species complexes, where at least one individual species will have appropriate environmental conditions irrespectively of prevailing weather conditions. Moreover, from the fungicide side it has to be considered that fewer fungicides with a new mode of action are released and increasing temperatures might also speed up resistance development of pathogens against fungicides. All these current and future problems will contribute to an over-riding importance of breeding. In future, wheat cultivars with durable multi-disease resistances are needed that are stable under extreme environmental conditions, such as heat and drought.

Gene transfer and genome editing would greatly help for those diseases where no native resistances are available in elite breeding materials (reviewed by Sánchez-Martin and Keller 2019). In particular, when new methods are applied that allow a faster cloning of genes than ever before (reviewed by Hatta et al. 2019), genome editing would be beneficial for a faster reaction of the breeders to a changing world, because time matters. In the short term, genomics-assisted resistance breeding will help to screen larger populations for the same costs and thus greatly accelerate breeding progress (Miedaner et al. 2020b). This will especially apply when the resistance donors are non-adapted to NW Europe and a genomics-assisted backcrossing and integration process is performed.

The question whether a reduction in disease resistance level due to changing environmental conditions was related to the weakening of the plant, the strengthening of the pathogen or a changed expression of a particular resistance gene, is difficult to answer. However, increasingly researchers try to solve this riddle using innovative and powerful analysis techniques in the laboratory. Preliminary results are available relating to temperature stress that show that specific resistance genes were influenced, but knowledge if water stress can affect the action of specific resistance genes is completely missing in wheat. Therefore, appropriate research approaches are needed including in-depth research on the effect of climate change on quantitative disease resistances. To summarize, wheat is an ideal model plant to continue research more in-depth; however, the focus should not remain mainly on yellow rust resistance genes and their temperature stress sensitivity, but should be extended to other economically important diseases, for example, leaf rust, stem rust, and FHB and should include water stress sensitivity as well, although complex interactions will likely occur.
